# Rapid In-Field Detection of Airborne Pathogens Using Loop-Mediated Isothermal Amplification (LAMP)

**DOI:** 10.3390/microorganisms12122578

**Published:** 2024-12-13

**Authors:** Alessia Bani, Corinne Whitby, Ian Colbeck, Alex J. Dumbrell, Robert M. W. Ferguson

**Affiliations:** 1 School of Life Sciences, University of Essex, Wivenhoe Park, Colchester, CO4 3SQ, UK; a.bani@derby.ac.uk (A.B.); cwhitby@essex.ac.uk (C.W.); colbi@essex.ac.uk (I.C.); adumb@essex.ac.uk (A.J.D.); 2 Aquatic Research Facility, Nature-Based Solutions Research Centre, University of Derby, Derby DE22 1GB, UK

**Keywords:** bioaerosol, airborne pathogen, rapid detection, loop-mediated isothermal amplification (LAMP), biological aerosols

## Abstract

Multiple human and plant pathogens are dispersed and transmitted as bioaerosols (e.g., *Mycobacterium tuberculosis*, SARS-CoV-2, *Legionella pneumophila*, *Aspergillus fumigatus*, *Phytophthora* spp., and *Fusarium graminearum*). Rapid, on-site methods to detect airborne pathogens would greatly enhance our ability to monitor exposure and trigger early mitigation measures across different settings. Analysis of air samples for microorganisms in a regulatory context is often based on culture-based methods, which are slow, lack specificity, and are not suitable for detecting viruses. Molecular methods (based on nucleic acids) could overcome these challenges. For example, loop-mediated isothermal amplification (LAMP) is rapid, sensitive, specific, and may detect microbial pathogens from air samples in under 60 min. However, the low biomass in air samples makes recovering sufficient nucleic acids for detection challenging. To overcome this, we present a simple method for concentrating bioaerosols collected through liquid impingement (one of the most common methods for bioaerosol collection). This method paired with LAMP (or other molecular approaches) offers simple, rapid, and sensitive detection of pathogens. We validated this method using three airborne pathogens (*Mycobacterium tuberculosis*, *Legionella pneumophila*, and *Aspergillus fumigatus*), and we were able to detect fewer than five cells in a 15 mL liquid impinger air sample in under 60 min. This simple method offers rapid pathogen detection without the use of specialist equipment, and it can be used across healthcare, education, environmental monitoring, and military settings.

## 1. Introduction

Bioaerosols are small biogenic aerosols ranging in size from ~0.05 to 100 μm, and they represent a significant public health risk [1,2,3] (For example, airborne cell fragments, endotoxins, allergens, metabolites, and other toxins can act as sources of irritation, leading to respiratory diseases, such as asthma and chronic obstructive pulmonary disease (COPD). Aerosolized microbial pathogens can act as transmission routes for infectious diseases (e.g., Legionnaires’ disease, tuberculosis, aspergillosis, COVID-19 [4,5]) or be used in bioterrorism [6]. However, our ability to quantify and mitigate the risks of bioaerosols is hampered by a lack of quality data on their distribution, composition, dispersal, and sources [1,2,3]. We also lack highly sensitive methods that can rapidly detect airborne pathogens in situ, which would enable real-time responses to outbreaks.

Conventionally, culture-based methods are used for regulatory monitoring of bioaerosols [7]. For example, these include the “M9—Environmental Monitoring of Bioaerosols at Regulated Facilities”, European Technical Specifications CEN TS 16115-1 and CEN TS 16115-21 [8,9,10]. Yet, culture-based methods are labor-intensive, they typically yield low sample throughput, and they cannot provide the rapid results required to support a flexible and immediate response [7]. Some pathogenic microorganisms are also less amenable to culturing or may require subsequent identification steps to confirm their identity [11]. Furthermore, current culture-based bioaerosol monitoring methods used in a regulatory context are generally unsuitable for the detection of airborne viruses (e.g., influenza, coronaviruses). Nucleic-acid-based (molecular) methods have transformed our understanding of the bioaerosol microbiome [12,13,14,15], with tremendous potential for their application across monitoring and regulatory contexts [7].

Public health officials require methods that are fast, simple to use, and provide robust results if they are to act rapidly and deploy mitigation strategies. Loop-mediated isothermal amplification (LAMP) can provide rapid (<60 min), sensitive, and robust identification of pathogens at the strain level (for bacteria, fungi, and even viruses). Furthermore, as it uses simple equipment (e.g., a simple heating block), it is relatively cheap, and it can be deployed across field settings [16,17,18,19,20,21]. However, applying this method to air samples that typically contain low biomass, low nucleic acid yields, and potentially high levels of inhibitory compounds (e.g., humic acids, inorganic particles) is challenging. Thus, standardised methods to collect bioaerosols for molecular analysis are still to be established, but they remain urgently needed [7,12,22,23].

**Figure 1:**
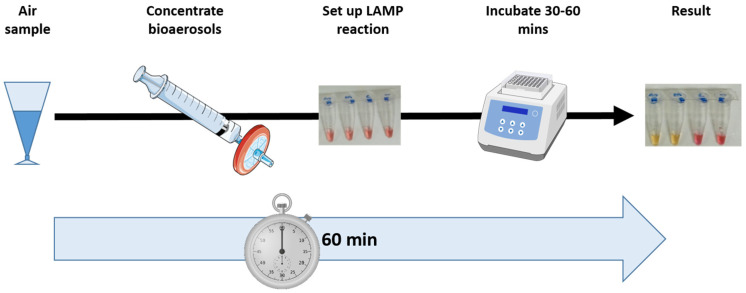
Workflow for rapid detection of airborne pathogens, from the air sample to the result in under 60 min.

As molecular methods are highly sensitive (as low as a single copy of the target gene for LAMP), the efficiency of any concentration steps used to overcome issues associated with lower biomasses is the key factor in determining test sensitivity. Liquid impingement is one of the most popular methods for bioaerosol sampling; it is familiar to regulators, and, importantly, it can collect sufficient biological material for molecular analysis in <20 min [12]. However, the bioaerosols are collected into a liquid medium of 10–15 mL (for example, Coriolis µ) and then require further steps to concentrate the sample. Thus, we present an optimized in situ sample concentration approach that works with any liquid impingement method commonly used for sample collection before screening with LAMP for specific pathogen detection. Our methods provide a simple, low-cost approach for sample concentration that can be analyzed using molecular methods for rapid (<1 h), specific (gene-level identification), and sensitive (one copy of the target gene) identification of airborne pathogens (Figure 1). Here, we present the experimental validation of these methods.

## 2. Materials and Methods 

**Sample concentration methods tested:** Three methods were tested for pathogen recovery from air samples that could be collected using any liquid impingement method. **Method 1:** Centrifugation of the sample at 13,400× *g* for 15 min, followed by removal of the supernatant and resuspension of the pellet in 20 µL of ultrapure water. A 5 µL aliquot of the resuspended pellet was added directly to the LAMP reaction. **Method 2:** Filtration of the sample with a 0.2 µM pore size cellulose acetate hydrophilic syringe filter (Minisart, Sartorius, Göttingen, Germany) and back flushing of the sample with 1 mL of ultrapure water. A 5 µL aliquot of the 1 mL elute was then added to the LAMP reaction. **Method 3:** Filtering 15 mL sample though a 47 mm polycarbonate filter with a 0.4 µm pore size (Cyclopore, Whatman, MA, USA, Fisher Scientific, Loughborough, UK) as recommended for air sampling by Ferguson et al. [12], and then punching out a 5 mm disk of the filter with a hole punch and placing the disk directly into the 50 µL LAMP reaction.

**Experiment 1: Evaluation of the suitability of LAMP for the detection of pathogens in air samples.** We evaluated four LAMP assays for the detection of *Escherichia coli* (*malB* gene) and three airborne pathogens (*Mycobacterium tuberculosis* (IS6110 gene), *Legionella pneumophila* (16S rRNA gene), and *Aspergillus fumigatus* (ancx4 gene) (see Table S1 for details)) without DNA extraction and at low concentrations (<100 target cells) within 60 min. Serial dilutions of each microorganism for the LAMP assay were prepared using molecular-grade ultrapure water (Thermo Fisher, Oxford, UK) to determine the limit of detection and the time required to detect it through a visible color change of the LAMP reaction mix. All reactions were carried out in triplicate with two technical replicates for each culture. For preparation of the serial dilutions and the PCR controls, *E. coli* DH5α was obtained from ATCC (p2B1862-1dhL-ara), and the target genes for the *M. tuberculosis*, *L. pneumophila*, and *A. fumigatus* assays where synthesized as double stranded DNA (Twist Bioscience) (Table S1). *Rhodococcus* sp. was obtained from the Essex University Culture collection. Primers to detect *E. coli* target the *malB* gene [24], for *M. tuberculosis* they target the IS6110 gene [25], for *L. pneumophila* they target the 16S rRNA gene (*L. pneumophila* [26], and for *A. fumigatus* they target the *ancx4* gene (*A. fumigatus* [27]) (Table S1). LAMP assays (colorimetric and florescent) were carried out as described below.

**LAMP assays:** LAMP assays used two pairs of primers, inner primers (FIP and BIP) and external primers (F3 and B3). A primer master mix was prepared at 10× final concentration in molecular-grade ultrapure dH_2_O (Thermo Fisher, Oxford, UK). Colorimetric LAMP mixtures (25 µL total unless otherwise stated) contained 5 µL of target DNA/cell mixture in a serial dilution from 10^5^ to the final 10^1^ concentration, 2.5 µL of 10× primer mix (final concentration 1.6 µM of each inner primer, 0.2 µM of each external primer, and 0.4 µM of each loop primer), and 12.5 µL of WarmStart^®^ Colorimetric LAMP 2X Master Mix (New England BioLabs, Hitchin, UK) (containing 0.8 mM MgSO_4_, 1 U Bst 2.0 WarmStart DNA polymerase, WarmStartRTx, Phenol Red for pH detection of LAMP, and X µM of each dNTP). LAMP reactions were performed at 65 °C, and changes in color were monitored starting from 30 min and thereafter every 10 min. Samples were chilled briefly to maximize the color change.

For fluorescent LAMP, a total of 25 µL was used: 5 µL of target DNA/cell mixture in a serial dilution from 10^5^ to the final 10^1^ concentration, 2.5 µL of 10× primer mix (to give a final concentration of 1.6 µM of each inner primer, 0.2 µM of each external primer, and 0.4 µM of each loop primer), 12.5 µL of WarmStart^®^ LAMP Kit (New England BioLabs, Hitchin, UK), 0.5 µL of dye, and 4.5 µL of PCR-grade water. Samples were run on a OptiGene machine (ProLab diagnostics, Round Rock, TX, USA) at 65 °C for at least 80 min, and melting curves were used to determine the specificity of the amplification (melting curves 65–95 °C increase of 0.5 °C). LAMP products were visualized through gel electrophoresis for 30 min at 100 volts (1.5% agar in Tris-acetate-EDTA (TAE) buffer) and stained with Ethidium bromide (1 µg/mL).

**Experiment 2: Validation of recovery of pathogens from an air sample**. To determine which of the three sample concentration methods proposed was most effective, between 10^1^ and 10^3^ *E. coli* cells were spiked into 15 mL of molecular-grade ultrapure water (Thermo Fisher, Oxford, UK). Recovery and colorimetric LAMP was performed as previously described. Results were checked using a 1.5% agarose gel. The syringe filter method was also performed for the *L. pneumophila* and *M. tuberculosis* LAMP assays. For these, the target genes were transformed into *E. coli* cells (as described below). Either 200 or 1000 cfu of the transformed cells containing the gene for either pathogen was added to 15 mL of water, which would give a theoretical final concentration of either 1 or 5 cells per 5 µL for each LAMP reaction tested. Culture-based analysis confirmed that the final cell concentration was 0.82 ± 0.37 for the theoretical 1 cell/5 µL reaction and 4.71 ± 1.58 cells per 5 µL for the theoretical 5 cells/5 µL reaction for *M. tuberculosis*. For *L. pneumophila,* it was 0.79 ± 0.33 and 22.35 ± 5 cells per 5 µL, respectively, for the theoretical 1 cell/5 µL reaction and 5 cells/5 µL reaction. For positive controls, 5 µL of ultrapure water containing cells as desired was added directly to the LAMP reaction.

**Transformation of *E. coli*:** Vectors (pTwist Amp High Copy, Twist Bioscience, South San Francisco, CA, USA) containing LAMP gene targets for all pathogens were synthesized by Twist Bioscience (USA) and resuspended in 1X TE buffer according to the manufacturer’s instructions. Transformation reactions were carried out using the high-efficiency *E. coli* competent cells TB095 kit (Promega, Madison, WI, USA) according to the manufacturer’s instructions (JM109 High efficiency >10^8^ cfu/µg, Promegea, Madison, WI, USA). For each transformation, cells were diluted either 1:10 or 1:100, and 100 µL of either undiluted or diluted cells was plated onto LB agar plates containing ampicillin (100 µg/mL) and incubated at 37° overnight. We validated that the transformed cells would still trigger a positive LAMP reaction by directly adding the cells to 5 µL of dH_2_O, which was then used in a colorimetric LAMP reaction. A positive reaction was obtained even when just one cell was present in the LAMP reaction. From a single colony, cells were transferred into liquid LB media containing ampicillin (100 µg/mL). Based on culture-dependent data (CFU), the number of cells in the original culture was quantified and diluted to achieve the desired cell concentration to test the limit of detection (LOD). The transformed cells were spiked into ~15 mL of sterile water (volume in accordance with the final concentration desired) and then filtered through a 0.22 µm PES filter. Then, 100 µL of the filtered water was plated onto LB agar plates containing ampicillin (100 µg/mL) and incubated at 37 °C for 48–96 h to determine if any cells passed through the filter. The cells were eluted from the filter using 1 mL of PCR-grade water and used in the LAMP colorimetric assay. Then, 100 µL was plated out as described above to determine the % cell recovery rate.

**Experiment 3: Validation of colorimetric LAMP in polluted air sample matrix.** To determine if the LAMP method is inhibited by contaminants present in air samples, air samples were collected from a service road under the University of Essex (Colchester, UK, 51.876116, 0.94554) using a Coriolis µ air sampler (Bertin instruments, Montigny-le-Bretonneux, France). The Coriolis µ air sampler used deionized water or PBS as the collection matrix (15 mL), and collection was for 20 min at 300 L/min (as recommended in Ferguson et al. [12] for the collection of biological material for molecular analysis). The collected samples were used to prepare the serial pathogen concentration dilution to be tested using LAMP, as previously described. All reactions were carried out in triplicate, with two technical replicates each.

## 3. Results and Discussion

### 3.1. Experiment 1: Evaluation of Suitability of LAMP for Detection of Pathogens in Air Samples

**Figure 2:**
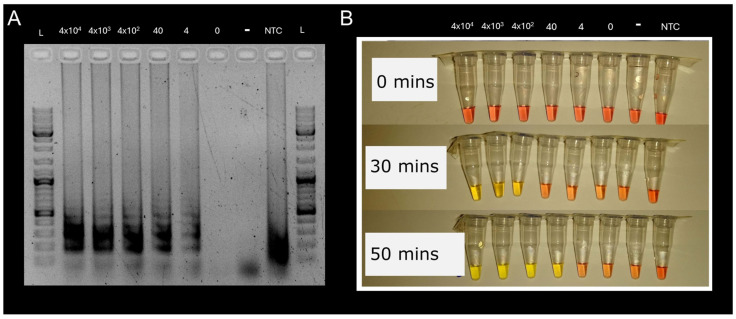
(**A**) Gel electrophoresis image of LAMP products and (**B**) reaction tubes at 0, 30, and 50 min for LAMP assay for the *E. coli malB* gene. Yellow color indicates positive LAMP reaction, orange indicates negative reaction. L = ladder (1 KB); the number indicates the number of cells in the reaction. *Rhodococcus* sp. negative control contained (10^4^ cells reaction^−1^). NTC = No template control (i.e., PCR-grade water in place of DNA template/cells).

**Evaluation of colorimetric and fluorescent LAMP with a non-pathogen:** We were reliably able to detect 40 *E. coli* cells per reaction with the *malB* gene LAMP assay (Table 1) by color change (40 min to detection) and fluorescent LAMP (46 min to detection) (Table 1, Figure 2). The negative control (10^4^ cells per reaction of *Rhodococcus* sp.) did not show a positive reaction through either color change or florescent methods. There was also no reaction for samples of the *E. coli* mixture when diluted to extinction (0 cells). It was possible to detect as few as four cells in a reaction mix through a color change but only after 90 min, but as there was also a color change in the NTC control (PCR-grade water in place of DNA template/cells); after 90 min, it would not be possible to distinguish this from a false positive. Gel electrophoresis analysis of the no template control (NTC) product showed a smear, in contrast to the “ladder effect” observed in the wells with positive LAMP reactions (Figure 2). This “ladder effect” results from the formations of concatemers of the DNA fragment between the F3 and B3 primers during successful LAMP reactions, whereas the smear likely results from primer dimer interactions [28]. Importantly, we were able to detect 40 cells of *E. coli* reliably without the need to isolate or purify nucleic acids, providing a very rapid detection method. However, DNA extraction may have further improved sensitivity. A simple heating step may also increase sensitivity by releasing nucleic acids from more robust cells (e.g., fungi and Gram-negative bacteria) [29].

**Evaluation of colorimetric and fluorescent LAMP for pathogen assays:** For *M. tuberculosis,* it took 50 ± 8.16 min for a positive reaction at the lowest concentration of target gene copies (10^1^ copies per reaction). The highest concentration (10^5^ copies per reaction) gave a color change in 40 min (Table 1). The melt temperature for the assay was 90.66 ± 0.83 °C. For *L. pneumophila,* at the higher concentration (10^5^ copies per reaction), the time it took to change the color was the same as the *M. tuberculosis* assay (i.e., 40 min), with a melt temperature of 87.23 ± 0.43 °C (Table 2). For the lowest concentration (i.e., 10^1^ copies per reaction), the time to a color change was slower at 63.33 ± 12.47 min. There was always positive amplification with 10^2^ copies per reaction (63.33 ± 9.43 min), giving a slightly higher effective detection limit (Table 2). *A. fumigatus* was the slowest, taking 73 ± 9.43 min for a color change at 10^1^ copies reaction and 50 min at the highest concentration (10^5^ copies per reaction) with a melt temperature of 90.61 ± 0.48 °C.

These results confirm the utility of LAMP for airborne pathogen detection. Positive reactions were achieved even with 10 gene copies in the reaction mix for *M. tuberculosis* and *A. fumigatus*, and detection of *L. pneumophila* was reliable at 100 cells per reaction, giving ample sensitivity when pairing this with the low biomass in air samples. Although not tested here, RT-LAMP can be performed with a simple one-step reaction using the same reagents, so detection of key airborne viral pathogens should also be possible, including SARS-CoV-2 and Norovirus [30,31].

A key limitation of LAMP is dimer formation, which results in false positives. For all of the primer sets tested, dimer formation only resulted in a false positive after ~80 min. Therefore, when the concentration of the target gene is low (i.e., <10^1^ copies per reaction), it may be difficult to distinguish between the actual positive results and the false positives (e.g., Table 1). One option to overcome this issue would be the use of LAMP reactions with loop primers to speed up positive reactions and differentiate them from the false positives caused by dimer formation (e.g., [32]). The use of a secondary confirmation test (which is conventionally required for any diagnostic test) would also improve confidence in detection, even at lower concentrations. Here, we used the melt peak temperature as a confirmation test (see also [33,34]) and/or gel-electrophoresis. Neither method, however, is suitable for a rapid in situ test, as both require specialist equipment, and performing agarose gel electrophoresis will increase the time taken to achieve validation. Alternatively, an oligo-probe-based test could be applied to confirm the formation of the correct product, and this could be achieved with a lateral flow test kit requiring no specialist equipment (for example, see [25,35]). Direct sequencing of LAMP products on-site using Oxford Nanopore technology would also be possible (e.g., LamPore, [36,37]). Not only could this be used to confirm positive detections of airborne pathogens, but it could also allow for the analysis of SNPs, which could provide additional information on evolutionary epidemiology to determine the sources, dispersal, and evolution of an airborne pathogen in real time.

### 3.2. Experiment 2: Validation of Recovery of Pathogens from an Air Sample

As previously discussed, the key determinant of sensitivity in this method is the concentration of bioaerosols from an air sample for molecular analysis. As described in the methods, we tested three methods to concentrate microorganisms from a liquid impingement air sample. **Method 1:** centrifugation. **Method 2:** filtration and back flushing. **Method 3:** direct addition of a 5 mm disk from a filter to a LAMP reaction. The most effective method was Method 2 (filtration of the collection fluid from the air sampler through a 0.2 µm syringe filter, followed by back flushing with 1 mL of ultrapure water to recover cells), giving a detection rate of 85 ± 12% with 10^1^ cells and a 100% detection rate with >10^2^ cells (Table 3). Centrifugation was only effective when samples where spiked with >10^2^ cells, but recovery was poor for lower concentrations (10^1^ detection rate of 30 ± 12%, Table 3). Method 3 (directly using a 5 mm section of filter spiked with cells in the LAMP reaction) was not effective at any concentration, with detection rates lower than 50% even with 10^3^ cells and detection rates of 5 ± 10% for 10^2^ cells and 0% for 10^1^ (Table 3). Both filter-based methods (Methods 2 and 3) have the advantage that they can be used with air filtration directly.

Liquid impingement was previously shown to require a shorter time than air filtration to collect sufficient material for molecular analysis, therefore making it the ideal methodology for rapid detection of airborne pathogens [12]. However, as air filtration provides greater total yields of DNA per m^3^ of air sampled (albeit at a slower rate, [12]), it may be useful in some contexts. For example, this includes fixed location pathogen surveillance (in a classroom or a hospital ward) or measuring long-term personal exposure to pathogens, such as by nurses or military personnel.

We further tested recovery with syringe filters (Method 2) with *E. coli* cells transformed with plasmids containing the target genes for LAMP detection of the pathogenic bacteria (*M. tuberculosis* and *L. pneumophilia*). Culture-based analysis of the concentrated sample showed a recovery rate of 74.5 ± 11.9%, with a minimum of 61.3 ± 21.6% for *L. pneumophila* and maximum of 89.7 ± 12.2 for *M. tuberculosis* (Table 4). Culture-based analysis also verified that no cells were present in the filtrate, only in the back-flushed sample. The sensitivities of experiment 1 (Table 2) and experiment 2 (Table 4) were identical independently of whether the LAMP reaction was carried out on synthetic oligo or the target DNA was still inside of a microbial cell. However, the detection time if the target DNA was inside of a microbial cell was slower when compared to the synthetic oligo experiment. The reaction was slower for target DNA inside of the competent cell by three and seven minutes for *M. tuberculosis* and *L. pneumophila,* respectively (Table 4). Detection of one cell of *M. tuberculosis* occurred in 43.33 ± 7.45 min, and five cells were detected in 38.33 ± 3.72. Detection of one cell of *L. pneumophila* occurred in 45.5 ± 5, and five cells were detected in 47.78 ± 9.16 min (Table 4).

Here, we confirm that a simple method utilizing a syringe filter is suitable for the concentration of bioaerosols for pathogen detection using LAMP. The syringe filter method performed better than centrifugation (which is a method that requires specialist equipment), but it was included as we expected it to perform the best [12]. It has been previously reported that the recovery of some spores through centrifugation might be poor due to their buoyancy [38]. This may make centrifugation a poor choice for the detection of fungal pathogens regardless. However, here, we tested it on bacterial cells, and while the performance was by no means poor, it was surprisingly worse than for the filtration method, which had a lower theoretical concentration factor.

### 3.3. Experiment 3: Validation of Colorimetric LAMP in Polluted Air Sample Matrix

Many air samples contain high concentrations of contaminants that may inhibit the amplification of nucleic acids [12]. For example, these include fine particulates consisting of various materials (e.g., heavy metals, salts, volatile organic compounds, other inorganics, lipopolysaccharide, endotoxins and other aeroallergens, humic acids) [39]. When *M. tuberculosis*, *L. pneumophila*, or *A. fumigatus* DNA was spiked into PBS as the liquid matrix by the Coriolis µ (as recommended in [12]), the time taken for a color change increased for all pathogens by ~10 min for *M. tuberculosis* and *L. pneumophila* and 6 min for *A. fumigatus*. However, when water was used as the liquid matrix, in the “polluted air sample”, the matrix had no effect on the time to detection of any of the pathogens (in fact, it was marginally improved) (Table 5). These results show that there is minimal inhibition of LAMP in an environmental air-sampling matrix. This is not surprising, as LAMP is typically more robust to inhibition than other PCR-based methods [40]. However, this should remain a consideration during further development of downstream analysis of air samples.

### 3.4. Potential for Detection of Airborne Viruses

Due to the need for an appropriate host, culture-based methods are not practical for the detection or monitoring of airborne viruses. Furthermore, most of the crucial airborne viruses relevant to human health present in indoor air are RNA viruses and therefore require an extra step when detected using PCR-based methods (e.g., influenza, coronaviruses, respiratory syncytial virus (RSV)). As LAMP reagents typically include the ability to conduct reverse transcription and LAMP in one step, it is an ideal strategy for molecular virus detection (as demonstrated for SARS-CoV-2 by [21]). The crucial question is how best to recover viruses from the air. While many methods have been used for airborne virus sampling, no standardized methods currently exist [41,42]. Wet impingers, such as the Coriolis µ and the BioSampler (SKC), have been shown to be effective for sampling airborne viruses [43,44], and they are also compatible with our filter-based concentration method without modification. We therefore suggest that our method would be suitable for airborne virus detection.

### 3.5. Potential Limitations of the Method in an Operational Setting

While the sample concentration method presented here shows sensitivity down to a few gene copies or target cells when paired with LAMP, we do not know how it will perform in the field. One major limitation of using molecular analysis of air samples is the presence of contaminants that might inhibit PCR or LAMP [12]; we have tested this by using a sample matrix from a semi-urban environment, but we are not able to cover the full range of environmental contexts here, and further testing is required. This method has also not been tested on aerosolized agents, and given that the sensitivity reported here is as low as five target cells, the efficiency of the air sampling method itself is likely the key limitation and may require further optimization [7,44].

A key question is not simply what is the best sensitivity we can achieve but what threshold should we aim for. Typical concentrations of pathogens across different environments are largely unknown. Critically, there is a limited understanding of the relationship between airborne concentrations and exposure (inhalation) and the relationship between exposure and health outcomes [7]. For the pathogens used in this study, the ID_50_ for *M. turberculosis* in humans has been reported to be as low as <10 cells. The ID_50_ for *Legionella* spp. is unknown [45], while for *A. fumigatus*, acceptable levels in outdoor air are 500 CFU/m^3^ higher than in the background [8]. Given the sensitivity of the method reported here, it should be able to detect health-relevant concentrations of these agents, assuming the air sampling method is representative of or more efficient than the inhaled dose.

One limitation of the method reported here is its ability to scale for high-throughput sampling. Ideally, a large number of locations or individuals (in the case of personal sampling) could be monitored, but as this method relies on a manual step and relies on specialist air sampling equipment, it cannot be scaled up easily. Automation of sampling and concentration via a microfluidic chip (as reported by [46] for airborne *Staphylococcus aureus*) could represent a suitable strategy for high-throughput monitoring with LAMP if the addition of the concentrated sample to the LAMP reaction could also be automated. However, one major limitation of microfluidic air sampling is the low airflows achieved. For example, Jiang et al. (2016) [46] achieved a maximum flowrate of 79.2 mL/min, giving an estimated 16.63 L of air volume sampled after 3.5 h. This is in comparison with an established bioaerosol sampling method, such as Coriolis µ, that can sample 1000 L in 3.5 min, potentially giving much greater sensitivity. A further limitation of microfluidics is if the chip needs to be reused. Sterilizing fluidic channels between uses in order to prevent false positives is challenging and presents a particular issue with LAMP, which is typically far more sensitive than PCR. To overcome this issue, additional (potentially manual) steps in the methods would be required, and thus the potential to scale to high-throughput would be reduced or would require multiple single-use devices at additional cost.

## 4. Conclusions

Here, we optimized and validated a simple approach for air sample concentration that when paired with molecular methods provides an easy, rapid, and sensitive approach to detecting airborne pathogens anywhere. Our approach has flexibility in that it could combine multiple different molecular analyses, such as qPCR [47] and gene sequencing. However, by using LAMP, we show that this method is rapid (results in <1 h), specific (gene-level identification), and sensitive (one copy of the target gene or cell). Importantly, this method requires no specialist equipment (a simple syringe filter is used) other than for air sampling, making it relatively inexpensive, and it is deployable to monitor emissions, dispersal, and personal bioaerosol exposure across a variety of settings. For example, this could include quantifying personal exposure to airborne pathogens in healthcare and education settings, monitoring and warning of outbreaks of crop/livestock pathogens in agriculture, monitoring emissions of health-relevant pathogens from waste treatment (e.g., *A. fumigatus* from composting), or even quantifying exposure to deliberately aerosolized agents (for example, bioterrorism or military use).

## Figures and Tables

**Table 1:** Sensitivity and specificity of the LAMP assay for the *E. coli malB* gene (*n* = 3).

Cells Reaction^−1^	Time to Detection Based on Color Change (mins)	Time to Detection (mins) SYBR
4 × 10^4^	30	21
4 × 10^3^	30	24
4 × 10^2^	30	28
40	40	46
4	90	0
0	NA *	NA*
Negative control ^†^	NA *	NA*
NTC	Some change at 90	NA*

^†^ Negative control contained 10^4^ *Rhodococcus* sp. cells reaction^−1^; melt temperature for this reaction was 86.97 ± 0.44 °C. * NA denotes not detected.

**Table 2:** Time to LAMP reaction color change and the resultant effective limit of detection (LOD) at the highest (10^5^ gene copies) and lowest concentrations (10^1^ gene copies) for each pathogen (see Figure S1 for raw images).

Pathogen	Mean Time for Color Change (mins)		Effective LOD		Melt T (°C)
	Lowest Concentration	Highest Concentration	Time (mins)	Copies Reaction^−1^	
*M. tuberculosis*	50 ± 8.16	40 ± 0	50	10^1^	90.66 ± 0.83
*L. pneumophila*	63.3 ± 12.47	43.33 ± 4.71	55	10^1^	87.23 ± 0.43
*A. fumigatus*	73 ± 9.43	50 ± 0	73	10^1^	90.61 ± 0.48

Data are an average of *n* = 6 replicates each (triplicates with duplicate technical replicates each). The highest concentration is 10^5^ copies per reaction, while the lowest concentration is 10^1^ copies per reaction.

**Table 3:** Detection rate of *E. coli* cells recovered using various methods. For the control, *E. coli* cells were added directly to the reaction at the desired concentration.

	Detection Rate (%)		
Number of Cells in Sample	10^3^ Cells	10^2^ Cells	10^1^ Cells
Control ^†^	100%	100%	95 ± 10%
Centrifugation	100%	100%	30 ± 12%
Filtration and elution	100%	100%	85 ± 12%
Direct from filter paper	45 ± 25%	5 ± 10%	0%

^†^ Number of cells indicated was added directly to the reaction. Mean of 4 trials with *n* = 5 (total 20 per method).

**Table 4:** Detection rate using syringe filter recovery of *E. coli* cells transformed with plasmids containing the target genes for LAMP detection of either *M. tuberculosis* or *L. pneumophila* (See Figure S2 for raw images).

Target	Theoretical Cells in Reaction 5 µL	Real Cells in Reaction 5 µL	Time to Detection (mins)	Recovery Rate (%)
*M. tuberculosis*	1	0.82 ± 0.37	43.33 ± 7.45	70.33 ± 17.34
*M. tuberculosis*	5	4.71 ± 1.58	38.33 ± 3.72	89.68 ± 12.15
*L. pneumophila*	1	0.79 ± 0.33	45.5 ± 5	61.33 ± 21.64
*L. pneumophila*	5	22.35 ± 5	47.78 ± 9.16	77 ± 24.12

Data are an average of *n* = 6 replicates each (triplicate samples with duplicate technical replicates each).

**Table 5:** PBS air-polluted samples vs. water air-polluted samples (see Figures S3 and S4 for raw images).

	Mean Time for Color Change (mins)			
Pathogen	Phosphate-Buffered Saline (PBS)		Water	
	Lowest concentration ^†^	Highest concentration	Lowest concentration	Highest concentration
*M. tuberculosis*	48.33 ± 2.36	43.33 ± 4.71	36.6 ± 4.71	33.33 ± 4.71
*L. pneumophila*	60 ± 0	50 ± 21.60	46.67 ± 9.43	40 ± 0
*A. fumigatus*	50 ± 0	53.33 ± 4.71	56.67 ± 17	40 ± 0

Data are an average of *n* = 6 replicates each (triplicate samples with duplicate technical replicates each). ^†^ The highest concentration was 10^5^ copies per reaction and the lowest concentration was 10^1^ copies per reaction for both PBS and water testing.

## Data Availability

The original contributions presented in this study are included in the supplementary material. Further inquiries can be directed to the corresponding author.

## Supplementary Materials

The following supporting information can be downloaded at: https://www.mdpi.com/article/10.3390/microorganisms12122578/s1, Table S1. Primer sets used in this study. Figure S1. Colorimetric LAMP assay for experiment 1. Figure S2. Colorimetric LAMP assay for experiment 2. Figure S3. Colorimetric LAMP assay for experiment 3 PBS. Figure S4. Colorimetric LAMP assay for experiment 3 H_2_O.
