# Supplementary material for: Rapid In-Field Detection of Airborne Pathogens Using Loop-Mediated Isothermal Amplification (LAMP)
Source: Microorganisms. 2024 Dec 13;12(12):2578. doi: 10.3390/microorganisms12122578 (PMC11678261; doi:10.3390/microorganisms12122578)
Supplement: Supplementary file 1 [file microorganisms-12-02578-s001.zip › microorganisms-3296341-supplementary.pdf]

Table S1. Primer sets used in this study

Agent	Primer name	Sequence	qPCR/PCR limit of detection	LAMP limit of detection
M. tuberculosis (Kaewphinit 2013)	MT_F3	GCCAGATGCACCGTCGA	Not reported	5.2 gene target copies
	MT_B3	GACACATAGGTGAGGTCTGC		
	MT_FIP	AGCGATCGTGGTCCTGCGGTTTTGATGACCAAACTCGGCCTGT		
	MT_BIP	TCCCGCCGATCTCGTCCATTTTTACCCACAGCCGGTTAGGT		
L. pneumophila (Lu et al 2011)	LP_F3	CGTTACCCACAGAAGAAGC	5 pg of genomic DNA	5 pg of genomic DNA
	LP_B3	ACCCTCTCCCATACTCGA		
	LP_FIP	AGTAATTCCGATTAACGCTCGCAACCGGCTAACTCCGTGC		
	LP_BIP	GGCGTAAAGGGTGCGTAGGTGACCAGTATTATCTGACCGTCC		
A. fumigatus (Tang et al 2016)	AF_F3	AGATACGGACGACGAGGC	10^2 gene target copies	10 gene target copies
	AF_B3	TGACCGGGAATGTCCTCATC		
	AF_FIP	ATCTACGCGAGGCGAGGACGCTAGCGATCTGGAGCGTGAT		
	AF_BIP	GGTCCAGTTATGTCCGGGGCTGAAGCATGACGGGAACG		
E. coli (Hill et al 2008)	malB-F3	GCCATCTCCTGATGACGC	Not reported	<10 gene target copies
	malB-B3	ATTTACCGCAGCCAGACG		
	malB-FIP	CATTTTGCAGCTGTACGCTCGCAGCCCATCATGAATGTTGCT		
	malB-BIP	CTGGGGCGAGGTCGTGGTATTCCGACAAACACCACGAATT		

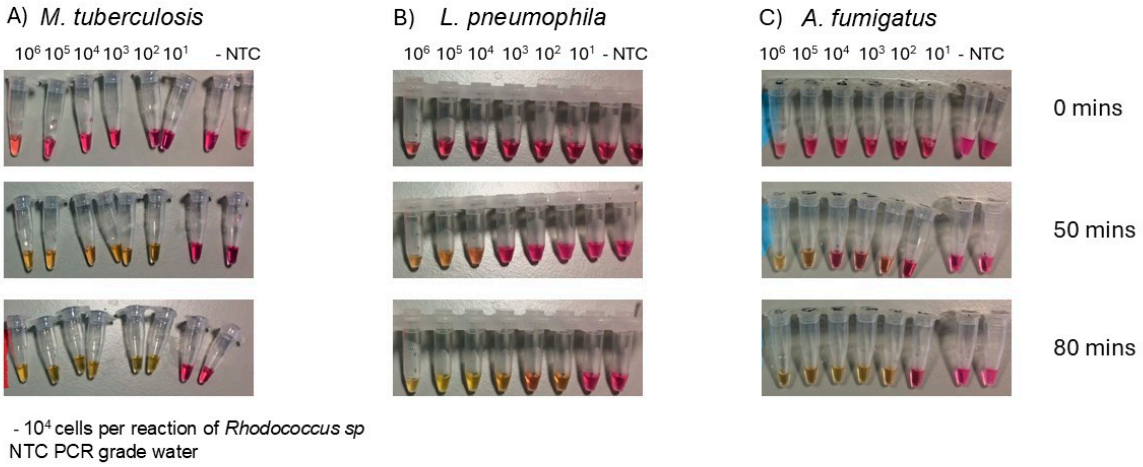

Figure S1. Panel A, B and C shown the colorimetric LAMP assay for experiment 1. For each pathogen, the same reactions are shown at 0, 50, and 80 minutes after incubation at 65°C. If the colour of the mix is yellow, there is a positive pathogen detection. In pink, if the reaction was not positive at the given time of the observation or in the case of negative control at all time as the pathogen DNA is not present in these samples.

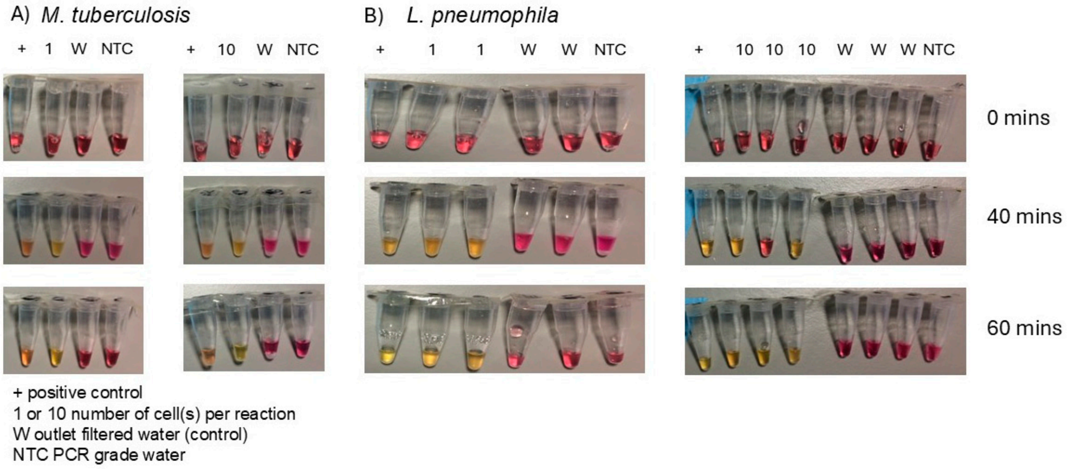

Figure S2. Panel A and B shown the colorimetric LAMP assay for experiment 2. For each pathogen, the same reactions are shown at 0, 40, and 60 minutes after incubation at 65°C. If the colour of the mix is yellow, there is a positive pathogen detection. In pink, if the reaction was not positive at the given time of the observation or in the case of negative control at all time as the pathogen DNA is not present in these samples.

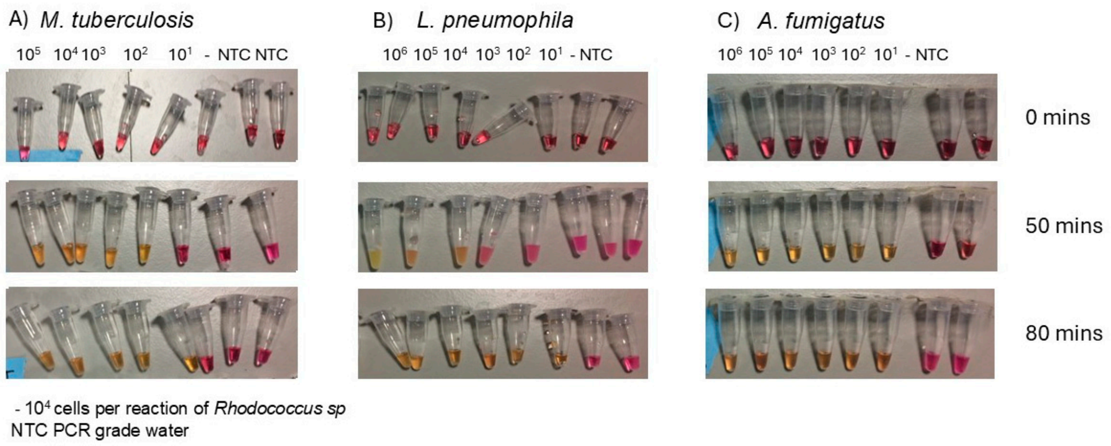

Figure S3. Panel A, B and C shown the colorimetric LAMP assay for experiment 3 PBS. For each pathogen, the same reactions are shown at 0, 50, and 80 minutes after incubation at 65°C. If the colour of the mix is yellow, there is a positive pathogen detection. In pink, if the reaction was not positive at the given time of the observation or in the case of negative control at all time as the pathogen DNA is not present in these samples.

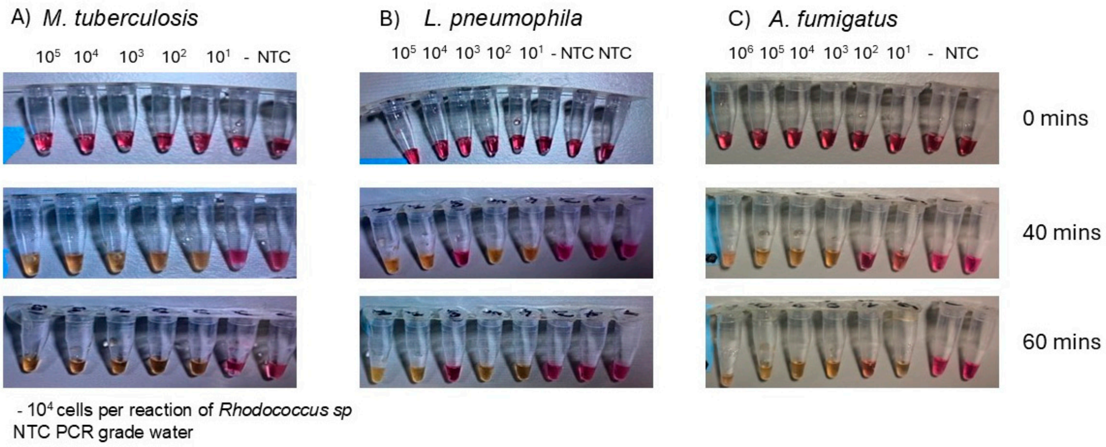

Figure S4. Panel A, B and C shown the colorimetric LAMP assay for experiment 3 H_2O. For each pathogen, the same reactions are shown at 0, 40, and 60 minutes after incubation at 65°C. If the colour of the mix is yellow, there is a positive pathogen detection. In pink, if the reaction was not positive at the given time of the observation or in the case of negative control at all time as the pathogen DNA is not present in these samples.
